# A direct comparison of the KB™ Basecaller and *phred *for identifying the bases from DNA sequencing using chain termination chemistry

**DOI:** 10.1186/1756-0500-3-257

**Published:** 2010-10-08

**Authors:** Richard W Hyman, Hui Jiang, Marilyn Fukushima, Ronald W Davis

**Affiliations:** 1 Stanford Genome Technology Centre, 855 S. California St., Palo Alto, CA 94304, USA; 2 Department of Biochemistry, Stanford University Medical School, Stanford, CA 94305, USA; 3 Department of Statistics, Stanford University, Stanford, CA 94305, USA; 4 Department of Genetics, Stanford University, Stanford, CA 94305, USA

## Abstract

**Background:**

Relatively recently, the software KB™ Basecaller has replaced *phred *for identifying the bases from raw sequence data in DNA sequencing employing dideoxy chemistry. We have measured quantitatively the consequences of that change.

**Results:**

The high quality sequence segment of reads derived from the KB™ Basecaller were, on average, 30-to-50 bases longer than reads derived from *phred*. However, microbe identification appeared to have been unaffected by the change in software.

**Conclusions:**

We have demonstrated a modest, but statistically significant, superiority in high quality read length of the KB™ Basecaller compared to *phred*. We found no statistically significant difference between the numbers of microbial species identified from the sequence data.

## Background

DNA sequencing by DNA polymerase chain termination was introduced by Sanger *et al*. [1] in 1977. In this technology, sequence is determined from the lengths of the terminated DNA chains. Electrophoresis is employed to separate the chains based upon length. A different fluorescent dye is covalently attached to each of the four dideoxy chain terminators. The presence of the dyes significantly affects the electrophoretic mobility of the chains. Therefore, sophisticated software must be employed to deconvolute the fluorescent signals into bases.

For some years, the suite of software of choice for DNA sequencing was introduced by Green and associates in 1998: *phred *for calling the bases in sequence reads, *phrap *for assembling the reads into contigs, and *consed *for displaying the contigs for editing [2-4]. Relatively recently, the manufacturer of the sequencing equipment, Applied Biosystems (ABI, Foster City, CA), introduced its own base calling software, the KB™ Basecaller, to replace *phred *http://www3.appliedbiosystems.com/cms/groups/mcb_marketing/documents/generaldocuments/cms_040412.pdf.

In our published study [5], we identified the microbes in the healthy adult human vagina by PCR amplifying the 16S ribosomal RNA genes, sequencing the genes with dideoxy chemistry, and identifying the microbes by comparison of the sequence to the data in the Ribosomal Database Project (RDP) [6]. We were concerned that the change in base-calling software would change the microbes identified. Primarily for this reason, we undertook a direct comparison of the KB™ Basecaller and *phred*, despite the fact that the comparison would be, and was, human labor intensive.

## Results

### Comparison of the high quality read lengths

**Figure 1:**
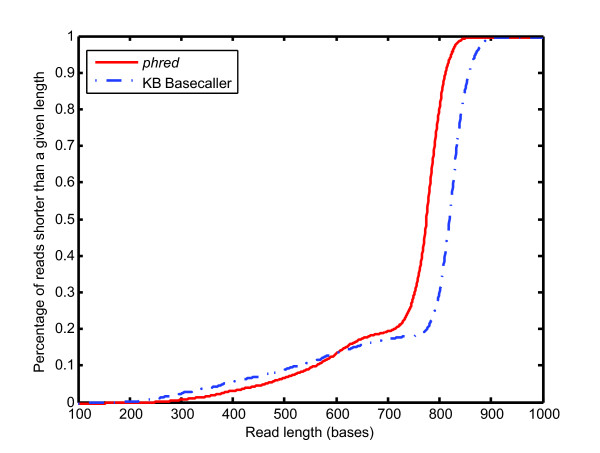
**High quality read length: *phred vs*. the KB™ Basecaller**. The red solid curve and blue dashed curve give the empirical distribution function of sequence read length produced by *phred *and the KB™ Basecaller, respectively. Sequence reads with fewer than 100 contiguous high quality bases have been discarded as failed reads.

The first comparison is of the high quality segments of the sequence reads derived from using *phred *to call the bases compared to the high quality segments of the sequence reads derived from using the KB™ Basecaller to call the bases. The results for all reads available for assembly are shown in Figure 1. We calculated the mean good quality read lengths for two cases: (1) sequence reads that were composed of a minimum of 100 consecutive high quality bases, because that is our minimum acceptable read length, and (2) reads that were composed of, at least, 500 good quality bases, because the longer the high quality segment, the more straightforward the assembly and the more secure the contig. These comparisons are shown in Table 1. For the first comparison (high quality read length > 100 bases), the KB™ Basecaller produced a mean read length of 763 bases with a standard deviation of 149 bases (n = 9,586), while *phred *produced a mean read length of 731 bases with a standard deviation of 116 bases (n = 9,572). Using the two sample t-test [7,8], we concluded that the reads produced by the KB™ Basecaller were, on average, statistically significantly longer than the reads produced by *phred *(p-value < 10^-15^). For the second comparison (> 500 high quality bases), the KB™ Basecaller produced a mean read length of 804 bases with a standard deviation of 74 bases (n = 8,717), while *phred *produced a mean read length of 756 bases with a standard deviation of 71 bases (n = 8,911). Again, the reads produced by the KB™ Basecaller were, on average, statistically significantly longer than the reads produced by *phred *(p-value < 10^-15^). Thus, in agreement with a poster on the ABI website, on average, the KB™ Basecaller yields longer high quality segments than *phred *yields http://www3.appliedbiosystems.com/cms/groups/mcb_marketing/documents/generaldocuments/cms_040383.pdf. However, whereas the poster states that the KB™ Basecaller produces high quality segments an average of ~ 100 bases longer than *phred*, we find that the average difference is 30-to-50 bases. An unknown amount of this difference may be due to two different methods for determining the length of a high quality segment.

**Table 1:** Mean high quality read length: *phred vs*. the KB™ Basecaller.

	KB™ Basecaller		*phred*	
	> 100	> 500	> 100	> 500
mean	763	804	731	756

SD*	149	74	116	71

No. of reads**	9586	8717	9572	8911

*SD, standard deviation. **No., number.

### Microbe identification comparison

As an example of the sequence data processed to microbe species identification, the data for Project 95 are shown in Tables 2, 3, 4. Data for the other four projects are in the Additional files [Additional files 1, 2, 3, 4, 5, 6, 7, 8, 9, 10, 11, 12]. In all five projects, there are modest differences between the current microbe identifications and number of supporting reads compared to our published study [5]. We ascribe these differences to improvements in software and the data added to the RDP since 2005.

**Table 2:** Project 95: Microbes by genus.

Closest named bacterium	KB™ Basecaller		*phred*	
	Number of reads	% of total reads	Number of reads	% of total reads
*Lactobacillus*	1781	98.2	1584	87.9

*Pseudomonas*	4	0.2	4	0.2

*Staphylococcus*	2	0.1	2	0.1

**Table 3:** Project 95: *Lactobacillus *by species.

Closest named bacterium	KB™ Basecaller		*phred*	
	Number of reads	% of total reads	Number of reads	% of total reads
*L. iners*	693	38.2	665	36.9

*L. crispatus*	606	33.4	448	24.8

*L. jensenii*	453	25.0	455	25.2

*L. sp*.	26	1.4	12	0.7

*L. fornicalis*	3	0.2	4	0.2

**Table 4:** Project 95: Novel bacteria.

Closest named bacterium	KB™ Basecaller		*phred*	
	Number of reads	% match	Number of reads	% match
*Lactobacillus*	19	78-97	74	84-97

uncultured	7	N/A	139	N/A

uncultured	0	N/A	13	89-95

Six statistical comparisons were performed employing the Wilcoxon signed-rank test [9,10]. The first comparison is of the total number of sequence reads in the five edited assemblies. The p-value is 0.1875. Thus, there is no statistically significant difference in the number of reads in the assemblies as produced by *phred *or the KB™ Basecaller. The second comparison is of the total number of microbial species identified. The p-value is 0.0625, which is the smallest p-value one can get using the Wilcoxon signed-rank test on five paired samples. While *phred *produced more species than the KB™ Basecaller in all five projects, this difference is not statistically significant.

Bacterial species from the genus *Lactobacillus *are the most common microbes found in the healthy adult vagina [*e.g*., [11]]. One of the five patients [Additional file 2, Supplemental Table S6] had (virtually) no *Lactobacillus *in her vagina, so the following comparison is among four projects. Consolidating all *Lactobacillus *species into the genus *Lactobacillus*, we compared the number of reads supporting the presence of *Lactobacillus *(p-value = 0.125). Thus, there is no statistically significant difference in the number of reads supporting the presence of *Lactobacillus*. A comparison of the number of reads supporting the presence of various *Lactobacillus *species (p-value = 0.125) and the number of different *Lactobacillus *species (p-value = 0.125) also failed to be statistically significant.

Conventionally, if the match of the sequence of the 16S ribosomal RNA gene to the closest sequence in the RDP is less than 97%, the microbial species is designated as novel [*e.g*., [12,13]]. For our last comparison, we compared the number of reads supporting the presence of novel species and the number of novel species. Again, *phred *produced more reads and more novel species than the KB™ Basecaller in all five projects, but, as both p-values are 0.0625, there is no statistically significant difference.

## Discussion

We have demonstrated a modest, but statistically significant, superiority in high quality read length of the KB™ Basecaller compared to *phred*. We found no statistically significant difference between the number of species identified from the sequence data processed starting with either software. Overall, it is gratifying that the two base-calling software led to the identification of the same microbes: *i.e*., microbe identification (our end product) is not a function of the base-calling software employed to call the bases from the raw sequence data.

## Conclusions

We have demonstrated a modest, but statistically significant, superiority in high quality read length of the KB™ Basecaller compared to *phred*. We found no statistically significant difference between the numbers of microbial species identified from the sequence data.

## Methods

We took the raw dideoxy sequence data from the last five women in our published study on vaginal microbes [5]. For the purposes of this comparison, we arbitrarily called them Projects 95-99. These sequences had already been deposited in the GenBank database [accession nos. AY958774-AY959212]. There are ~2,000 sequence reads for each woman. We started with the raw sequence data. In the first case, we called the bases with *phred *(incorporated in *consed *v14) [2]. A high quality base has a *phred *score (or equivalent) of 20 or higher [3]. In the second case, we called the bases with the KB™ Basecaller (v1.2; ABI, Foster City, California, USA). Standard default parameters were used for the two algorithms. It is possible that different results might have been achieved if different parameters had been employed. The accuracy of quality value assignment has not been examined. Any inaccuracies for either algorithm will have a direct impact on the average quality-trimmed read length. Thereafter, the sequence reads for both cases were processed in parallel with the same software. The sequence of each read was compared to the sequence of the plasmid vector. Plasmid bases were turned into "X"s. Because the plasmids had been grown in *E. coli*, the remaining sequence was compared to the sequence of *E. coli *DNA. By this process, a very few reads were removed from each dataset. Then, the reads were assembled into contigs by *phrap *[2,3], and the contigs were displayed in *consed *[4]. Every contig in every one of the (now) ten projects was edited by hand and, for consistency, by the same person. There were three major types of manual edits. For (virtually) every recombinant plasmid, there was a forward read and a reverse read [5]. (1) Some contigs were composed of only forward (or reverse) reads. We call these "half contigs". For each read in a half contig, the opposing read was found and brought into the contig. (2) In some cases, the forward read was in one contig and the reverse read was in a different contig. The appropriate read of the pair was moved. (3) At a given position, some reads had high quality base "X" while other reads had high quality base "Y". The one contig was split into two contigs. *Consed *provides a consensus sequence for each contig [4]. For microbe identification, the contig consensus sequences were compared to the data in the RDP (release 10) [6].

## Abbreviations

N/A: not applicable; No.: number; RDP: Ribosomal Database Project; SD: standard deviation.

## Authors' contributions

RWH conceived the comparison of the two base-calling software, hand edited all contigs, and wrote the manuscript. HJ performed the statistical evaluations of the data. MF employed the *phred *and ABI KB™ Basecaller software to call the bases, assembled the sequence reads with *phrap*, and displayed the contigs in *consed*. MF also compared the contig consensus sequences to the data in the RDP to identify the microbes and constructed the tables of microbes identified. RWD provided the intellectual, physical, and financial milieu for these experiments. All authors have read and approved the final manuscript.

## Supplementary Material

Additional file 1 **Table S5: Project 96: Microbes by genus**. A table showing the microbes identified and the number (percent) of their supporting reads for Project 96. Click here for file

Additional file 2 **Table S6: Project 96: *Lactobacillus *by species**. A table showing the *Lactobacillus *species identified and the number (percent) of their supporting reads for Project 96. Click here for file

Additional file 3 **Table S7: Project 96: Novel bacteria**. A table showing the novel species identified, the closest named bacteria, and the number (percent) of their supporting reads for Project 96. Click here for file

Additional file 4 **Table S8: Project 97: Microbes by genus**. A table showing the microbes identified and the number (percent) of their supporting reads for Project 97. Click here for file

Additional file 5 **Table S9: Project 97: *Lactobacillus *by species**. A table showing the *Lactobacillus *species identified and the number (percent) of their supporting reads for Project 97. Click here for file

Additional file 6 **Table S10: Project 97: Novel bacteria**. A table showing the novel species identified, the closest named bacteria, and the number (percent) of their supporting reads for Project 97. Click here for file

Additional file 7 **Table S11: Project 98: Microbes by genus**. A table showing the microbes identified and the number (percent) of their supporting reads for Project 98. Click here for file

Additional file 8 **Table S12: Project 98: *Lactobacillus *by species**. A table showing the *Lactobacillus *species identified and the number (percent) of their supporting reads for Project 98. Click here for file

Additional file 9 **Table S13: Project 98: Novel bacteria**. A table showing the novel species identified, the closest named bacteria, and the number (percent) of their supporting reads for Project 98. Click here for file

Additional file 10 **Table S14: Project 99: Microbes by genus**. A table showing the microbes identified and the number (percent) of their supporting reads for Project 99. Click here for file

Additional file 11 **Table S15: Project 99: *Lactobacillus *by species**. A table showing the *Lactobacillus *species identified and the number (percent) of their supporting reads for Project 99. Click here for file

Additional file 12 **Table S16: Project 99: Novel bacteria**. A table showing the novel species identified, the closest named bacteria, and the number (percent) of their supporting reads for Project 99. Click here for file
